# Platelet-Derived Extracellular Vesicles Promote Tenogenic Differentiation of Stem Cells on Bioengineered Living Fibers

**DOI:** 10.3390/ijms24043516

**Published:** 2023-02-09

**Authors:** Ana L. Graça, Rui M. A. Domingues, Manuel Gomez-Florit, Manuela E. Gomes

**Affiliations:** 1 3B’s Research Group, I3Bs—Research Institute on Biomaterials, Biodegradables and Biomimetics, Headquarters of the European Institute of Excellence on Tissue Engineering and Regenerative Medicine, University of Minho, AvePark, Parque de Ciência e Tecnologia, Zona Industrial da Gandra, Barco, 4805-017 Guimarães, Portugal; 2 ICVS/3B’s–PT Government Associate Laboratory, 4805-017 Guimarães, Portugal; 3 Health Research Institute of the Balearic Islands (IdISBa), 07010 Palma, Spain

**Keywords:** extracellular vesicles, platelets, stem cells, tenogenic differentiation, hierarchical scaffolds, composite living fibers

## Abstract

Tendon mimetic scaffolds that recreate the tendon hierarchical structure and niche have increasing potential to fully restore tendon functionality. However, most scaffolds lack biofunctionality to boost the tenogenic differentiation of stem cells. In this study, we assessed the role of platelet-derived extracellular vesicles (EVs) in stem cells’ tenogenic commitment using a 3D bioengineered in vitro tendon model. First, we relied on fibrous scaffolds coated with collagen hydrogels encapsulating human adipose-derived stem cells (hASCs) to bioengineer our composite living fibers. We found that the hASCs in our fibers showed high elongation and cytoskeleton anisotropic organization, typical of tenocytes. Moreover, acting as biological cues, platelet-derived EVs boosted the hASCs’ tenogenic commitment, prevented phenotypic drift, enhanced the deposition of the tendon-like extracellular matrix, and induced lower collagen matrix contraction. In conclusion, our living fibers provided an in vitro system for tendon tissue engineering, allowing us to study not only the tendon microenvironment but also the influence of biochemical cues on stem cell behavior. More importantly, we showed that platelet-derived EVs are a promising biochemical tool for tissue engineering and regenerative medicine applications that are worthy of further exploration, as paracrine signaling might potentiate tendon repair and regeneration.

## 1. Introduction

Tendons are connective tissues that link muscles to bones, enabling body movement. They are composed of a dense, anisotropic, and multihierarchical organized extracellular matrix (ECM) that mainly consists of collagen type I. This unique architecture provides tendons with high mechanical strength and resilience [1]. Tendon diseases, which can include injury, overuse, inflammation, infection, and genetics, produce swelling, tenderness, pain, and stiffness. In severe cases, tendons can rupture, leading to more serious complications [2]. Worldwide, tendon disorders are one of the main causes of disability that decrease the quality of life of individuals across the demographic spectrum and represent a substantial economic burden on society [3,4]. At present, the principal treatments used for tendon injuries are not able to fully restore tendon functionality, and because of the hypovascular and hypocellular nature of tendons, they present a reduced healing capacity, which also limits the success of the available therapies [5,6].

In recent years, tissue engineering strategies combining biomaterials, biological signals, and cells have been proposed as promising therapeutic tools to achieve complete tendon regeneration and tendon function re-establishment [6]. Typically, biophysical and biochemical cues that mimic the native structure and microenvironment of the tendon are used to induce cells to acquire a tenogenic phenotype. These cues include, among other things, the mechanical stimulation of the cells, the presence of certain growth factors in the microenvironment, and the development of three-dimensional (3D) scaffolds that provide support and guidance for the cells [7,8]. For example, the use of anisotropic fiber-based scaffolds that recreate tendon topography on a nano-to-macro scale have been demonstrated to foster stem cell commitment to the tenogenic phenotype [9,10,11,12,13]. In addition, the combination of these approaches with biological cues, such as platelet-derived growth factor (PDGF), transforming growth factor-beta (TGF-β), or tropoelastin, can guide and support cells in situ to maintain the tenogenic phenotype [14,15,16,17,18]. More recently, the concept of composite living fibers, consisting of biomaterial cores coated with hydrogels laden with cells, has been explored to mimic tendon tissue structure [19]. Previous studies have shown that various load-bearing biomaterials coated with hydrogels can sustain cell survival, migration, and alignment [20,21,22]. Additionally, in order to introduce biofunctionality, core biomaterials have been coated with human platelet lysate, which enhanced stem cells migration and tenogenic differentiation [9,23].

Increasingly, platelet-derived products are being viewed as a cost-effective source to obtain biomolecules with therapeutic potential [24]. Platelets play a central role in tissue homeostasis after injury by releasing a wide range of growth factors, cytokines, and other signaling biomolecules that are essential in the modulation of cellular responses to injury [24]. Platelet-derived products such as plasma-rich plasma (PRP) are currently utilized in tendon, ligament, and cartilage repair procedures, as well as in joint replacement surgery, as an inexpensive and autologous source of regenerative factors [25]. Nevertheless, the use of platelet-derived products in the clinical setting is still widely debated. While they are believed to possess therapeutic benefits, the lack of standardized preparation, the variable quality of donor samples, and the uncontrolled release of biomolecules, all remain major concerns. In light of these issues, further studies are needed to fully assess the safety and efficacy of this type of treatment [24,26].

The scientific community has been increasingly focusing on the study of the cellular secretome and the crosstalk that exists between cells. A major component of the secretome are extracellular vesicles (EVs), which are nanosized lipid bilayer particles secreted by cells. They contain different cargos that reflect their origin, such as RNA, proteins, and metabolites [27,28]. Actually, EVs have been combined with biomaterials as powerful biochemical cues to stimulate tissue repair and regeneration [26,29,30]. Previous studies have shown that MSCs-derived EVs combined with different scaffolds were able to promote tendon regeneration in animal models, potentially through increased stem cells proliferation, differentiation, and anti-inflammatory effects [31]. However, the production of EVs from MSCs has some limitations, resulting in low EV yields and reproducibility; thus, there is a need for alternative sources of EVs with therapeutic and regenerative potential [26,32].

In this study, we evaluated the potential of different platelet-derived EV populations as biochemical cues to promote stem cells tenogenic commitment on composite living fibers composed of a polycaprolactone (PCL) core and a collagen shell, envisioning future tendon tissue engineering and regenerative medicine applications. We used a 3D continuously aligned fibrous scaffold to mimic the tendon hierarchical architecture, which was coated with collagen in order to both mimic the ECM composition and to provide a hydrated matrix to allow the encapsulation of stem cells and entrapment of EVs. The biological performance and functionality of the system were evaluated using human adipose-derived stem cells (hASCs) encapsulated in the collagen-based hydrogel either with or without EVs from platelets. Specifically, we focused on assessing how different platelet-derived EV populations influence the behavior of hASCs and modulate the capacity of hASCs to differentiate towards the tenogenic lineage.

## 2. Results and Discussion

### 2.1. Platelet-Derived EVs’ Characterization

The use of EVs for tissue engineering and regenerative medicine has largely relied on mesenchymal stem cell (MSC) cultures to isolate them [33]. Nevertheless, the production of large amounts of high-quality EVs in an economically feasible way necessary for clinical translation requires further refinement [32]. Moreover, EVs are normally harvested from cell culture supernatants grown in 2D, whose physiological significance still remains unclear. In order to convey several of these limitations, we hypothesize that platelets are a potential and underexplored source of EVs with potential roles in tissue repair and regeneration with high clinical translation potential. Platelet EVs can be easily obtained from expired or surplus platelet units/concentrates produced in blood banks (10–25% of all platelet units) [34], which might be revalorized and reduce the production costs of EV-based products. Moreover, regulatory agencies have already approved some platelet-based products to expand cells for cell therapies [35].

**Figure 1:**
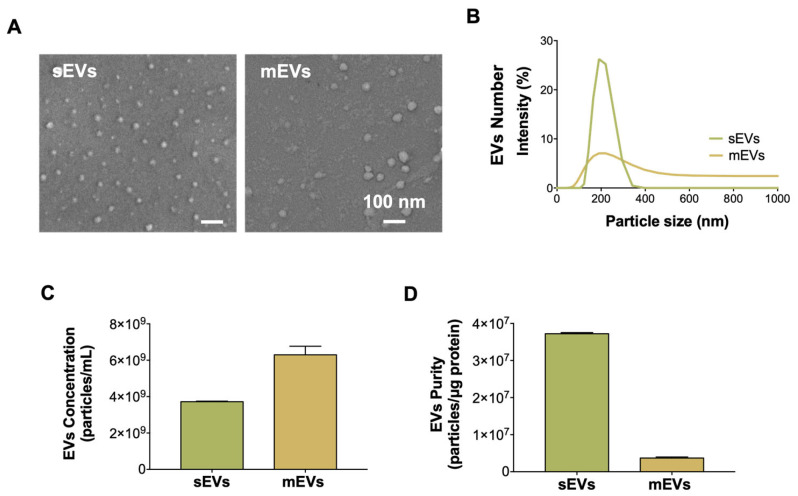
Characterization of platelet-derived EVs. (**A**) SEM images of sEVs and mEVs. Scale bar: 100 nm. (**B**) Representative size distribution of platelet-derived EVs measured using DLS. (**C**) EVs concentration determined by EXOCET assay. (**D**) EVs purity assessed by the number of vesicles to protein concentration ratio.

We isolated two distinct platelet-derived EV populations using different centrifugation speeds, namely small EVs (sEVs) and medium EVs (mEVs), as described and extensively characterized elsewhere [27]. SEM images revealed that the isolated nanovesicles had a spherical shape and intact membranes, which is characteristic of EVs (Figure 1A). In order to determine the size of the EVs, a DLS analysis was performed (Figure 1B). It showed that the sEVs tended to be smaller than the mEVs, showing average diameters of 270.1 ± 18.5 nm and 304.4 ± 38.4 nm, respectively. Despite the slightly bigger size of the EVs between the current and previous reports [27], it was likely attributable to the different methods used to measure them. Moreover, it can be observed that the sEVs’ population was more uniform, presenting a peaked unimodal size distribution, compared with the mEVs that showed a more heterogeneous and flattened size distribution (Figure 1B), which is also in agreement with previous findings [27]. Next, the number of EVs and their purity (ratio particles to protein) were assessed (Figure 1C,D). The graphs show a higher yield of the mEVs (63.0 × 10^9^ ± 4.7 × 10^8^ mEVs/mL) than the sEVs (37.2 × 10^9^ ± 2.9 × 10^7^ sEVs/mL) from the same starting volume, as expected from the centrifugation protocol. However, the purity of the sEVs was higher than the mEVs, which is also in line with our earlier results [27]. This was most likely due to the isolation method used for the sEVs, which involved high-speed centrifugation steps that removed sample contaminants [36].

### 2.2. Tendon-Mimetic Constructs Development and Characterization

The lack of reliable 3D models that recreate the hierarchical structure and microenvironment of tendons is one of the main obstacles faced by the scientific community in making further progress in this field [7,37]. Tendons are made up of a complex network of hierarchically organized collagen fibers, ranging from single nanofibrils to larger bundles. Tenocytes and stem/progenitor cells are located within the tendon ECM and are responsible for tissue homeostasis [38].

**Figure 2:**
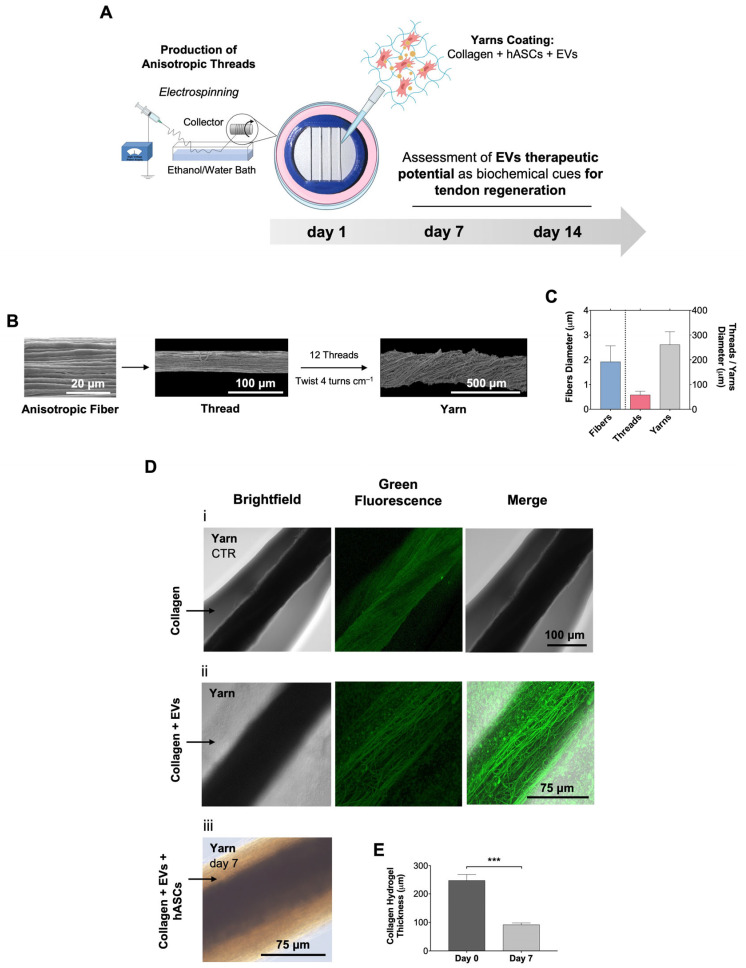
Design and characterization of 3D in vitro tendon-mimetic constructs. (**A**) Schematic illustration of the experimental set up used to develop tendon-mimetic constructs. Yarns were produced by electrospinning, placed in printed holders, and coated with a hydrogel consisting of collagen combined with EVs and hASCs. Then, the effect of EVs in hASCs was evaluated after 7 and 14 d of culture. (**B**) SEM micrographs of anisotropic fiber threads assembly into yarns of 12 threads followed by 4 turns/cm. Scale bars: 20 μm and 500 μm. (**C**) Diameter of anisotropic fibers, threads, and yarns. Data are presented as mean ± SD. (**D**) i—yarns coated with collagen (CTR); ii—yarns coated with EVs-collagen (SYTO; green); iii—yarns coated with EVs-collagen mixed with hASCs (day 7). Scale bars: 75 μm and 100 μm. (**E**) Assessment of collagen thickness from ii, at days 0 and 7 of culture. Data are presented as mean ± SD. Statistical differences: *** *p* < 0.001.

For this, we first aimed at developing a bioengineered 3D system to simultaneously recreate the physiological architecture and biological requirements of the native tendons (Figure 2A). We relied on previous works of our group to produce electrospun PCL fiber threads hierarchically assembled into yarns [9,10,14,39,40]. SEM micrographs (Figure 2B) allowed us to determine the diameter of the anisotropic fibers (1.9 ± 0.6 μm) constituting the threads (57.9 ± 15.4 μm), which were within the range of native collagen fibers (1–20 μm) [38]. Then, anisotropic fiber threads were assembled into yarns (260.8 ± 52.8 μm), mimicking the native hierarchical structure of tendon fascicles (150–1000 μm) (Figure 2C) [38,41]. The hierarchical structure of the tendon is responsible for its viscoelastic behavior while its longitudinal alignment guarantees that it can withstand high tensile forces [42]. PCL mechanical features were extensively evaluated in previous works, which showed that PCL nanofibrous scaffolds reached a tensile strength of 2.90 ± 0.41 MPa and a Young’s Modulus (stiffness) of 12.10 ± 1.26 MPa [39], meaning they were in the lower range of native tendon tensile properties (5–100 MPa and 20–1200 MPa, respectively) [43]. Moreover, besides the topographical features, an outer hydrogel layer consisting of collagen was added to the system to better reproduce the in vitro tendon microenvironment and to allow the subsequent encapsulation of cells and biological cues. As the main tendon ECM component, collagen has been widely used in the field of tendon tissue engineering to create 3D tissue-mimetic constructs suitable for cell seeding or encapsulation [7].

In our work, yarns were coated with a layer of bovine collagen to mimic the tendon ECM and to offer a hydrated niche for cell encapsulation and biomolecules entrapment (Figure 2D(i)). During the optimization, we found that the use of human collagen and additional collagen crosslinkers (e.g., human transglutaminase) caused excessive gelation that resulted in brittle hydrogels that broke apart from the yarns after removal from the holders (Figure S1, Supplementary Information). The living component of the composite fibers was introduced by encapsulating the hASCs in collagen-EVs hydrogel prior to gelling around the tendon-mimetic yarns (Figure 2D(iii)). Immediately after collagen gelation, a hydrogel layer with an average thickness of 247.3 ± 21.3 μm was observed (Figure 2E). Over the culture time (7 days), the hydrogel thickness significantly decreased to 91.7 ± 6.3 μm (Figure 2E). This was most likely due to the contraction of the collagen matrix by forces exerted by the encapsulated hASCs [44]. Additionally, this effect further supported contact guidance promoted by the cell’s closer interaction with the fiber’s surface topography, inducing cell elongation and a highly organized cellular orientation (Figure S2, Supplementary Information). The acquisition of an elongated cellular morphology with an aligned cytoskeleton is characteristic of tenocytes embedded in the tendon ECM and has been related to the tenogenic commitment of stem cells [45,46].

Next, in order to introduce the biological cues in the system, platelet-derived EVs were loaded within the collagen solution before coating the yarns. Using fluorescently labelled nanovesicles and confocal microscopy, we could observe that EVs were homogeneously distributed into the collagen hydrogel despite the autofluorescence of PCL (Figure 2D(ii)). Interestingly, although the EVs were thought to be secreted exclusively into a liquid phase, recently, the role of the ECM in regulating their sequestration and interactions with target cells has been described [47,48]. Therefore, the collagen-based hydrogel coating from our bioengineered living fibers, apart from providing a hydrated biomimetic environment adequate for cell encapsulation, may add further complexity to the system, interacting with EVs and locally regulating their presentation to target cells, which is an advantage compared with typical culture media supplementation. Moreover, EVs can be used as biological cues to broaden the design of bioengineered living fibers and allow for the introduction of biofunctional molecules into various natural and synthetic polymers-based hydrogels, such as alginate or methacrylated gelatin [20], instead of just limiting it to one type of biofunctional hydrogel shell, such as those previously reported based on platelet lysate [9,23]. In this way, the number of potential applications is greatly increased.

In conclusion, we successfully bioengineered composite living fibers made of hierarchically organized PCL electrospun fibers, coated with collagen, and encapsulating hASCs and biofunctional EVs. These fibers provide an in vitro system for tendon tissue engineering, allowing for the study of the tendon microenvironment and the influence of EVs on stem cell behavior.

### 2.3. Interaction of hASCs with Tendon Bioengineered Fibers and Platelet-Derived Evs

**Figure 3:**
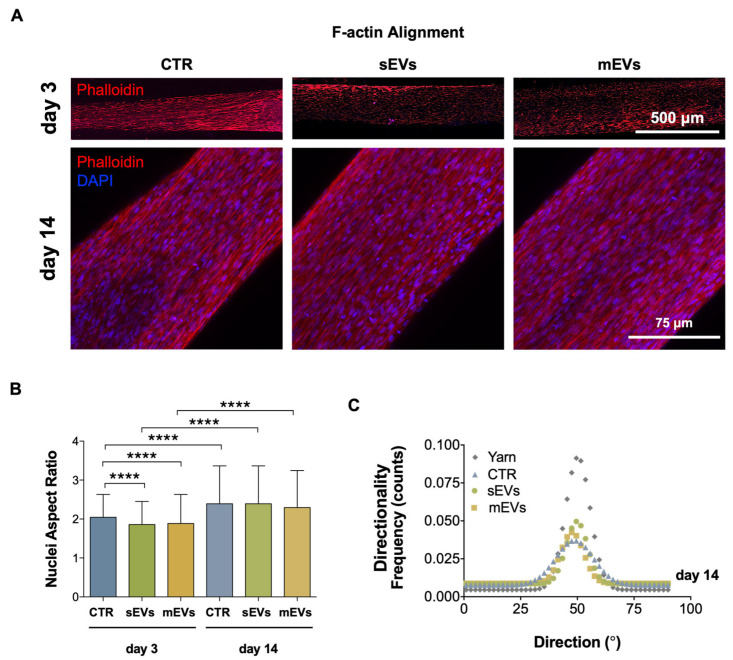
Morphometric analysis of hASCs onto collagen-coated biomimetic yarns either with or without EVs. (**A**) Confocal microscopy images of hASCs encapsulated in collagen-coated yarns after 3 d of culture (phalloidin: red) and 14 d of culture (phalloidin: red; DAPI: blue). Scale bars: 500 μm and 75 μm. (**B**) Nuclei aspect ratio of hASCs at 3 and 14 d of culture. Data are presented as mean ± SD. Statistical significance: **** *p* < 0.0001. (**C**) Directionality analysis of hASCs F-actin filaments after 14 d.

We used hASCs due to their high proliferation potential, easy access and isolation, low immunogenicity, low risk of tumor formation, and high potential in the regenerative medicine field. Samples with encapsulated cells were initially assessed by confocal microscopy after 3 d of culture to determine whether yarns or platelet-derived EVs influenced the hASCs’ morphology or alignment. Figure 3A shows that the hASCs on all groups were elongated and spread along the yarn length, following the yarns’ aligned topography. The elongation and alignment of the hASCs was evaluated by calculating the nucleic aspect ratio and performing a directionality analysis on the F-actin filaments after 3 and 14 d of culture. A high nuclei aspect ratio, indicating an elongated cell morphology [49], revealed that the fibroblast-like shape typical of tenocytes was quickly adopted by the hASCs in all groups, although some statistical differences between the control and EV samples were found (Figure 3B). After 14 d of culture, the spindle shape in the hASCs was more pronounced, with no significant difference between the groups (Figure 3B). The cell cytoskeleton directionality analysis showed that the hASCs aligned with the yarn topography, which was similar in the EVs and in the control groups after 14 d of culture (Figure 3C). These results suggest that the biophysical features of the yarns may be the primary factor influencing the hASCs’ morphology and alignment. Actually, cells can react to topographical and biological signals, acquiring a tenocyte-like morphology, which could have a positive impact on stem cell tenogenic differentiation, as previously shown [8,50].

### 2.4. Influence of Platelet-Derived EVs in hASCs’ Tenogenic Commitment

**Figure 4:**
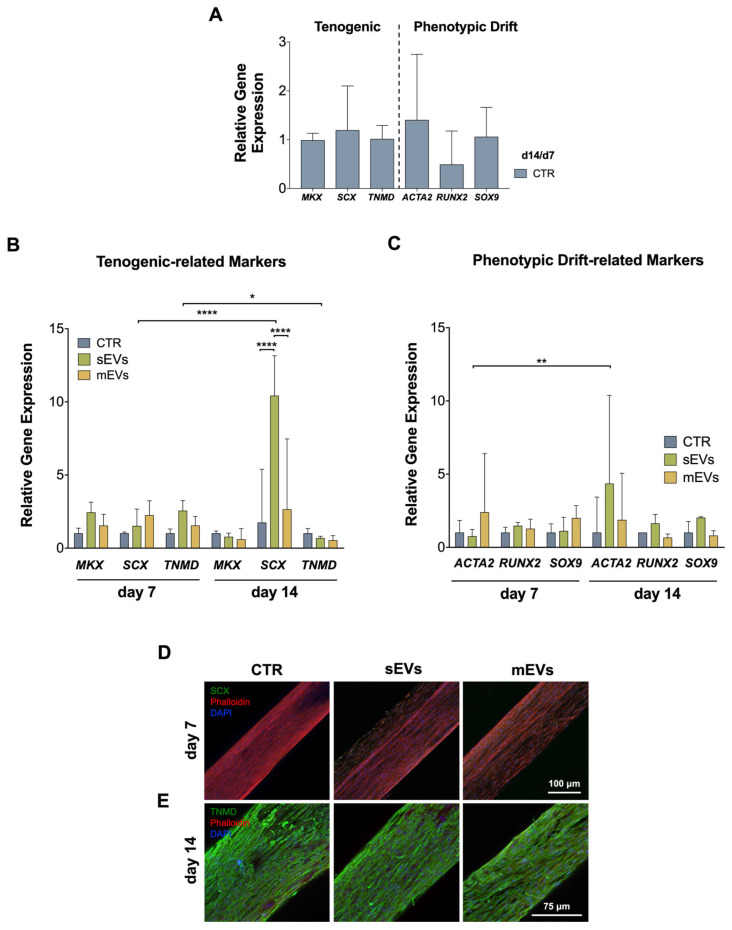
EVs effect on phenotypic-drift of hASCs encapsulated in yarns. (**A**) Control day 14/control day 7 ratio of tendon markers. (**B**) Gene expression analysis of tenogenic markers MKX, SCX, and TNMD, and (**C**) tenogenic drift markers ACTA2, RUNX2, and SOX9 after 7 and 14 days of culture. Gene expression results are presented as fold changes with respect to the control group at each time point. Statistical significance: * *p* < 0.05, ** *p* < 0.01, **** *p* < 0.0001. (**D**) Representative confocal images of immunolabelled samples against SCX (green) after 7 d and (**E**) against TNMD (green) after 14 d of culture. Images were counterstained with DAPI (cells nuclei, blue). Scale bars: 75 μm and 100 μm, respectively.

The tenogenic differentiation of the hASCs was first studied on control yarns (no EVs) through the analysis of the gene expression of key tenogenic markers such as scleraxis (*SCX*) and mohawk (*MKX*), two transcription factors expressed in early tendon development, and tenomodulin (*TNMD*), a transmembrane protein expressed by mature tenocytes [51,52]. As shown in Figure 4A, our bioengineered living fibers appear to support the tenogenic differentiation of stem cells over time, as indicated by the sustained expression of tenogenic markers. Furthermore, we also evaluated the gene expression profiles of phenotypic drift markers, namely, the myofibroblast differentiation marker alpha-smooth muscle actin (*ACTA2*), preosteogenic marker runt-related transcription factor 2 (*RUNX2*), and chondrogenic marker SRY-box transcription factor (*SOX9*). We observed that the expression of these markers tended to decrease over time (Figure 4A), also indicating a potential commitment of stem cells towards the tenogenic lineage.

Next, we evaluated how the expression of tenogenic and phenotypic drift markers in the hASCs was influenced by the platelet-derived EVs loaded within the collagen shell of the bioengineered living fibers. The expression of the tendon markers was upregulated on day 7 in the presence of EVs, compared to the control group, although these differences were not statistically significant (Figure 4B). Remarkably, a significant upregulation of *SCX* from day 7 to day 14 was observed, especially in the sEVs group, when compared to the control and mEVs groups, possibly indicating maintenance and maturation over the tenogenic phenotype [53]. Furthermore, the low levels of expression of genes related to tendon cells’ phenotypic drift were downregulated or sustained over the time of culture in the presence of EVs (Figure 4C), indicating that the nanovesicles did not potentiate to the hASCs’ differentiation into other lineages [54,55]. These results might be possibly related to the high content of PDGF and TGF-β in platelet EVs [27,40,56], which have been shown to enhance the tenogenic phenotype [14,15,16,17,18].

In addition, the expression levels of *ACTA2*, a myofibroblast differentiation marker, were upregulated over time in the presence of the sEVs (Figure 4C), which is in line with our previous findings using tendon-derived cells [40]. Interestingly, myofibroblasts’ activation is necessary to induce ECM synthesis, although their persistent activation leads to pathological fibrosis and tissue stiffening, which can impair organ function [44]. Therefore, we hypothesize that an early expression of *ACTA2* followed by a decrease might be beneficial in initiating ECM deposition and preventing later excessive myofibroblast activation. The tenogenic commitment of the hASCs in the presence of platelet-derived EVs was further evaluated by the immunostaining of SCX and TNMD after 7 and 14 d of culture, respectively. Figure 4D shows that the SCX expression was mainly located around the nuclei of the cells in all the control and EVs groups. On the other hand, the expression of TNMD was more cytoplasmic yet similar under all conditions (Figure 4E). Overall, the gene expression analysis revealed that the platelet-derived EVs promoted the tenogenic differentiation of the hASCs while preventing their phenotypic drift, despite immunostaining not revealing major differences.

### 2.5. Effect of Platelet-Derived EVs on ECM Contraction, Synthesis, and Remodeling

**Figure 5:**
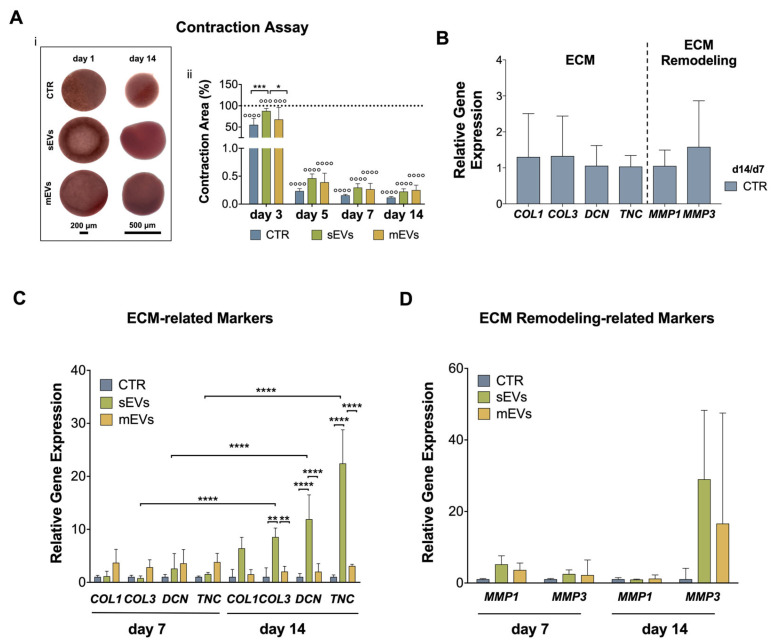
EVs’ role on matrix deposition by hASCs encapsulated in yarns. (**A**) Collagen contraction assay. i*—*representative pictures of collagen hydrogel loaded with hASCs either with or without EVs over time. Scale bars: 200 μm and 500 μm. ii*—*collagen gel contractility area after 3, 7, and 14 days, respective to day 1. Data are presented as mean ± SD. Statistical differences: * *p* < 0.05, *** *p* < 0.001, °°° *p* < 0.001, °°°° *p* < 0.0001 (° against day 1). (**B**) Control day 14/control day 7 ratio of ECM markers. (**C**) Gene expression of ECM deposition markers COL1A1, COL3A1, DCN, and TNC, and (**D**) ECM turnover markers MMP1 and MMP3 after 7 and 14 days. Target genes were normalized to the reference genes GADPH and GUSB. Gene expression results are presented as fold changes with respect to the control group at each time point. Statistical significance: ** *p* < 0.01, **** *p* < 0.0001.

In order to show any potential effects of platelet-derived EVs on the ability of the hASCs to remodel the ECM, we performed a gel-contraction assay. For this, drops of a collagen solution containing hASCs either with or without EVs were placed in a culture plate. After the formation of a hydrogel, the samples were followed for 14 d (Figure 5A(i)). We observed that the control gels (no EVs) contracted 45%, whereas the gels with the sEVs and mEVs contracted only 13% and 33%, respectively, after 3 d of culture (Figure 5A(ii)). Previous studies have indicated that the increased contractility of hASCs is linked with enhanced myofibroblast differentiation and fibrosis as well as the development of a proangiogenic phenotype that stimulates endothelial sprouting [44]. Therefore, platelet-derived EVs might have the potential to reduce pathologic fibrosis and vascularization associated with tendon diseases [7] and to promote the acquisition of a healthier tenogenic phenotype. Despite the fact that there was a significant contraction between day 3 and day 5 of culture for all groups, the contraction of the gels in the presence of EVs was lower than in the control group, but it still did not reach statistical significance (Figure 5A(ii)). Nevertheless, myofibroblasts are also responsible for ECM deposition and are key effectors in homeostasis restoration and tissue remodeling after injury [57]. Therefore, although these results might seem contradictory with the increased gene expression of *ACTA2* in the presence of EVs (Figure 4C), many factors affect the cell contractility, such as collagen microarchitecture and stiffness, which may have been affected by a newly deposited or remodeled ECM.

Additionally, to study the influence of platelet-derived EVs on the capacity of hASCs to synthesize and remodel the ECM, the gene expression of a range of tendon matrix markers was evaluated. On control samples (no EVs), the expression of the tendon ECM components, including collagen type 1 (*COL1A1*), collagen type 3 (*COL3A1*), the glycoproteins decorin (*DCN*), and tenascin (*TNC*), decreased over time (Figure 5B). On the contrary, the expression of ECM-remodeling enzymes, such as metalloproteinases −1 and −3 (*MMP1* and *MMP3*), was significantly upregulated from day 7 to day 14 of culture (Figure 5B). The addition of platelet-derived EV populations, specifically the sEVs group, significantly upregulated the expression of *COL3A1*, *DCN*, and *TNC* after 14 d of culture (Figure 5C). Moreover, the expression of *COL1A1* also increased in the sEVs group, although no statistical differences were found (Figure 5C). These results might also be related to an upregulation by EVs-derived TGF-β, a potent stimulator of ECM synthesis [58]. The early expression of these markers is typical of tendon tissue fibrillogenesis, thinning, and alignment [10,38,42]. Furthermore, *MMP1* and *MMP3* expression was increased in both EV groups compared with the control, but no significant differences were found (Figure 5D). Thus, the increased expression of tendon ECM-related markers, and the increased levels of *MMP3* after 14 d might indicate that the hASCs’ matrix is remodeling to a physiological tendon-like matrix [58,59].

Overall, small differences were observed when comparing the effects of the platelet-derived sEVs and mEVs on the hASCs. However, the results generally indicated that the sEVs had a superior effect than the mEVs on the hASCs’ tenogenic commitment. For example, the gene expression of the tenogenic marker *SCX* at day 14 (Figure 4B) was higher for the sEVs compared to the mEVs, as was the expression of the marker *ACTA2* (Figure 4C). This same pattern was also seen for the expression of two ECM markers, *COL3A1* and *TNC* (Figure 5C). These results might be connected to the high TGF-β1 content present in platelet-derived EVs [40]. TGF-β1 is known to enhance the tenogenic differentiation of stem cells, promote ECM synthesis, and upregulate the expression of *ACTA2* [60]. However, the mEVs were found to possess a higher concentration of TGF-β1 than the sEVs [40], and a higher effect of the mEVs would be expectable. Nonetheless, in the present work, we found that the purity of the sEVs was higher than the mEVs (Figure 1D), suggesting that the amount of TGF-β in the platelet-derived EVs preparations should be correlated to the purity of the EVs’ preparations. Moreover, it has been reported that TGF-β signaling is subjected to negative feedback regulation [61]. Future research should focus on this pathway to validate this hypothesis.

Although we did not delve into the mechanism of interaction between EVs and the cells, the results showed that the collagen-based hydrogel was able to deliver the platelet-derived EVs to the encapsulated hASCs. Interestingly, previous works using collagen membranes have demonstrated that EVs bind specifically to collagen via the EVs’ surface molecules, such as integrins, and are retained within the collagen matrix for prolonged delivery [62,63], which should be further investigated in our future works. These collagen membranes and fibrin hydrogels have been used to deliver stem-cell-derived EVs to improve tendon regeneration in vivo [31,64,65]. However, these hydrogels lack the physical cues provided by the anisotropic nanofibrous scaffolds underneath the collagen layer in our system.

In previous works conducted by our group, we found that the platelet-derived mEVs enhanced the formation of tube-like structures using endothelial cells while the sEVs were able to modulate macrophages’ polarization [27]. Moreover, using a tendon disease 3D model, we found that the sEVs promoted the recovery of the tendon cells’ healthy phenotype and the expression of tendon-related markers, while the mEVs showed potential anti-inflammatory effects [40], which is in line with the present work. In summary, these results suggest that platelet-derived EVs may be used as a promising source of biomolecules with complementary roles in the context of tendon tissue engineering. Future works using in vivo models should be performed to validate these findings.

## 3. Materials and Methods

### 3.1. Platelet-Derived EVs’ Production and Isolation

The platelet concentrate (PC) from a pool of 80–100 healthy donors was subjected to three freeze/thaw cycles, and the EVs were extracted from the lysates via differential centrifugation as described previously [27]. The PC was provided by Serviço de Imunohemoterapia do Centro Hospitalar de São João (CHUSJ, Porto, Portugal) under ethical commission approval (No. 363/18). The use of PC prepared from large pools of donors has been shown to standardize the composition of the obtained platelet-derived products [66]. Briefly, the mEVs were isolated after two centrifugations at 2000× *g* for 30 min at 4 °C, followed by centrifugation at 12,000× *g* for 45 min at 4 °C (FA-45-6-30 rotor; Eppendorf 5810 R, Eppendorf, Germany). The resultant supernatant was ultracentrifuged at 110,000× *g* for 2 h at 4 °C (S52-ST rotor, ThermoFisher Scientific, Sorvall Mx 120 Plus, Micro-ultracentrifuge, Waltham, MA, USA) to obtain the sEVs.

### 3.2. EVs’ Characterization

The nanoparticle size distribution was measured via dynamic light scattering (DLS) using a Malvern Zetasizer Nano ZS (Malvern Instruments, MAL1017850, Malvern, UK). The EVs’ structure was visualized using high-resolution scanning electron microscopy (SEM; Auriga CompactLV, Zeiss, Jena, Germany). For this, the EVs were fixed in 2.5% glutaraldehyde (Sigma-Aldrich, St. Louis, MO, USA) in phosphate-buffered saline (PBS, Sigma-Aldrich, St. Louis, MO, USA) followed by an ascending series of ethanol dehydration (50, 70, 90 and 100%) onto a glass substrate. To quantify the number of EVs, the EXOCET Exosome Quantitation Assay Kit (System Biosciences, Palo Alto, CA, USA) was used, according to the manufacturer’s instructions (n = 3). It is an antibody-free, colorimetric assay based on the quantification of the activity of Acetyl-CoA Acetylcholinesterase, an enzyme enriched in most EVs [67], which includes calibration standards to enable the calculation of the number of EV particles. Moreover, the EVs’ total protein was quantified using a microBCA protein assay kit (ThermoFisher Scientific) according to the manufacturer’s protocol. An extensive analysis of the physicochemical characteristics and composition of the EV populations was performed previously [27].

### 3.3. Production and Characterization of Anisotropic Yarns

An electrospinning solution was prepared by dissolving 17% poly-ε-caprolactone (PCL, average MW 80,000, Sigma-Aldrich, St. Louis, MO, USA) in chloroform (Honeywell, Charlotte, NC, USA) and *N*′-dimethylformamide (DMF, Carlo Erba Reagents, Val de Reuil, France) solution in a 7:3 ratio. A customized electrospinning setup was used to produce anisotropic fiber threads. A syringe filled with the electrospinning solution fitted with a 21 G needle was electrospun in a grounded water/ethanol bath (8:2) under a constant flow rate of 1.0 mL/h and voltage of 8.0–9.0 kV. Continuous anisotropic fiber threads were collected at 16 cm from the bath surface through a roller set at 20 cm from the needle at a constant speed of 6 cm/s. The temperature was maintained at 21–23 °C with a relative humidity of 43–45%. The produced anisotropic fiber threads were further assembled into yarns by grouping together 12 threads and twisting them at four turns per cm, as described in [10]. The yarns were then placed in 3D polylactic acid (PLA) holders designed using AutoCAD (version 2019) and printed using a B2X300 printer (Beeverycreative, Ílhavo, Portugal). These structures supported four yarns with an approximate length of 2.5 cm, and four-channel supports were produced to coat each fiber separately, as described in [9,40].

SEM (JSM-6010 LV, JEOL, Tokyo, Japan) operating at an accelerating voltage of 10 kV was used to assess the thread topography. Prior to imaging, all the samples were coated with gold under a vacuum for one minute (Cressington, UK). Random yarns (n = 3) were selected to measure the fiber diameter (n = 50 from each yarn) and alignment (directionality analysis) (n = 3) using the ImageJ 1.520 (NIH, Bethesda, MD, USA) software.

### 3.4. hASCs Isolation and Culture

The hASCs were harvested from liposuction aspirates as previously described in [68]. The samples were provided by the Department of Plastic Surgery of Hospital da Prelada (Porto, Portugal) through a cooperation agreement approved by the Ethical Committee of the University of Minho (reference 014-2019). Briefly, the tissue sample was rinsed in PBS with 1% antibiotic/antimycotic (A/A, Sigma-Aldrich, St. Louis, MO, USA) and then digested in 0.05% collagenase type I A (Sigma-Aldrich, St. Louis, MO, USA) solution for 1 h at 37 °C with agitation. The digested tissue was centrifuged at 800× *g* for 10 min at 4 °C to pellet the hASCs, which were expanded in a complete culture medium consisting of a minimum essential medium alpha (α-MEM, Life Technologies, Carlsbad, CA, USA) supplemented with 10% fetal bovine serum (FBS, Life Technologies, Carlsbad, CA, USA) and 1% A/A and used at passage 2–5.

### 3.5. Bioengineered In Vitro 3D Model

First, the 3D holders with anisotropic yarns were sterilized in 70% ethanol for 30 min, followed by 30 min of incubation in ultraviolet (UV) radiation. Subsequently, the yarns were coated with 100 μL of a solution containing 40% collagen (PureCol EZ Gel solution, 5074, Sigma-Aldrich, St. Louis, MO, USA) and 60% α-MEM supplemented with 10% FBS and 1% A/A with a density of 2.5 × 10^5^ hASCs/mL (control samples). Furthermore, the yarns were also coated with 40% collagen and 60% α-MEM supplemented with 10% of EV-depleted FBS (ThermoFisher Scientific, Waltham, MA, USA), 1% of A/A, 2.5 × 10^5^ hASCs/mL, and 100 μg/mL of the sEVs or mEVs (samples). After coating, the system was incubated for 1 h at 37 °C under humidified conditions to allow the gelation of the collagen solution. After gelation, a complete culture medium consisting of α-MEM supplemented with 10% FBS and 1% A/A was added to the yarns in the six wells containing the control samples, and α-MEM supplemented with 10% of EV-depleted FBS and 1% A/A was added to the samples containing EVs in the coating solution. The bioengineered system was maintained in culture for 14 days, and the medium was changed every two days. Moreover, the morphology of the collagen hydrogel was visualized by optical microscopy (DM750, Leica, Wetzlar, Germany), and its thickness was analyzed with ImageJ 1.520 (NIH, Bethesda, MD, USA) software (n = 4) immediately after gelation and 7 days later. Additionally, prior to the microscopy visualization, the EVs encapsulated in the collagen hydrogel were labeled with a SYTO RNASelect Green Fluorescent Cell Stain (Life Technologies, Carlsbad, CA, USA) according to the manufacturer’s instructions. The aspect ratio was calculated from these images (n = 4) by dividing the length by the width of at least 150 cells’ nuclei per group.

### 3.6. Collagen Contraction Assay

To assess the cell contractility in the collagen hydrogel, an equal volume of collagen solution with 2.5 × 10^5^ hASCs/mL and 100 μg/mL of the sEVs and mEVs (control samples without EVs in the solution) were cultured for 14 days. Contractility was assessed by the fractional decrease in the gel area on different pictures taken with a visible light inverted microscope over time (n = 6). Moreover, the contractility was normalized to the size of the collagen gel on day 1.

### 3.7. Immunofluorescence

Before staining, the yarns with the hASCs were fixed and permeabilized with 10% formalin (Bio-Optica, Milan, Italy) and 0.1% X-100 Triton (ThermoFisher Scientific, Waltham, MA, USA), respectively. All the samples were blocked with a 2.5% normal horse serum (Vector Laboratories, Burlingame, CA, USA) for 1 h at room temperature (RT). The samples were then immunostained using primary antibodies against antiscleraxis (SCX, rabbit, ab58655, 1:200, Abcam, Cambridge, UK) and antitenomodulin (TNMD, rabbit polyclonal, 1:200) generated against the TNMD C-terminus (237-317 aa) provided by Prof. Denitsa Docheva (produced in cooperation with Metabion International, Planegg, Germany, PAB 201603-00002), and they were incubated overnight at 4 °C. The samples were then incubated with 0.3% hydrogen peroxide (H_2_O_2_, PanReac AppliChem, Castellar del Vallès, Spain) for 15 min at RT and then incubated with Alexa Fluor 488 fluorescent secondary antibody (anti-rabbit, A21206, 1:200, ThermoFisher Scientific, Waltham, MA, USA) for 1 h at RT in the dark. Finally, the cell nuclei and cytoskeleton were counterstained with DAPI (1:1000; Biotium, Fremont, CA, USA) and phalloidin (1:500; Sigma-Aldrich, St. Louis, MO, USA), respectively, at RT for 30 min. The immunolabeled samples (n = 4) were analyzed using confocal laser scanning microscopy (CLSM, TCS SP8, Leica, Wetzlar, Germany).

### 3.8. RNA Extraction and Real-Time RT-qPCR

The total ribonucleic acid (RNA) was extracted from the system using the RNeasy Mini Kit (Qiagen, Hilden, Germany) according to the supplier’s instructions (n = 4) and was quantified using a nanodrop spectrophotometer (ND-1000, ThermoFisher Scientific, Waltham, MA, USA). A complementary deoxyribonucleic acid (cDNA) synthesis was performed using a qScript cDNA Synthesis Kit (Quantabio, Beverly, MA, USA) according to the manufacturer’s protocol using a Mastercycler Realplex (Eppendorf, Hamburg, Germany). The transcripts quantification presented in Table S1 (Supplementary Materials) was carried out via a real-time polymerase chain reaction (RT-qPCR) analysis using PerfeCTA SYBR Green FastMix kit (Quantabio, Beverly, MA, USA) in accordance with the kit instructions. Glyceraldehyde-3-phosphate dehydrogenase (*GAPDH*) and glucuronidase beta (*GUSB*) were used as reference genes. The relative expression was calculated using the ΔΔCt method [69]; it was first normalized against the reference genes and then against the average of the hASCs’ control samples at each time point.

### 3.9. Statistical Analysis

Statistical analyses were performed using GraphPad PRISM software (version 7.0) using default settings. The D’Agostino–Pearson test was performed to evaluate the normality distribution. An unpaired t-test (collagen thickness) or two-way analysis of variance (ANOVA) (collagen contraction, *MKX*, *COL1A1*, *SCX*, *ACTA2*, *SOX9*) was performed for normal distributions using the Bonferroni posthoc test for multiple comparisons. The Kruskal–Wallis test with Dunn’s correction was used for non-Gaussian distributions (aspect ratio, *DCN*, *TNC*, *COL3A1*, *TNMD*, *MMP1*, *MMP3*). Statistical significance was set at *p* < 0.05. The results are presented as mean ± standard deviation (SD).

## 4. Conclusions

In this work, we successfully bioengineered composite living fibers by combining anisotropic hierarchical fibrous structures that mimicked tendon organization with a collagen hydrogel coating to mimic the tendon microenvironment and to allow for the encapsulation of hASCs. Moreover, the platelet-derived EVs were incorporated within the hydrogel to provide biochemical cues to trigger tenogenic differentiation. These living fibers provided an in vitro system for tendon tissue engineering, allowing us to study not only the tendon microenvironment but also the influence of platelet-derived EVs on stem cell behavior. Overall, the results showed that the composite living fibers combined with the platelet-derived EVs’ modulated stem cell behavior, thereby enhancing the expression of crucial tendon markers, the production of a tendon-like ECM, and decreasing cell contractility. Furthermore, this study highlights the potential of platelet-derived EVs to improve the quality of tendon-like matrices generated by stem cells, providing a better understanding of their role in regulating the expression of tendon- and ECM-related components. Therefore, platelet-derived EVs are promising biochemical tools worthy of additional exploration, as paracrine signaling may potentiate tendon repair and regeneration.

## Data Availability

Not applicable.

## Supplementary Materials

The following supporting information can be downloaded at https://www.mdpi.com/article/10.3390/ijms24043516/s1.
