# Supplementary material for: Platelet-Derived Extracellular Vesicles Promote Tenogenic Differentiation of Stem Cells on Bioengineered Living Fibers
Source: Int J Mol Sci. 2023 Feb 9;24(4):3516. doi: 10.3390/ijms24043516 (PMC9959969; doi:10.3390/ijms24043516)
Supplement: Supplementary file 1 [file ijms-24-03516-s001.zip › ijms-2164392-supplementary.pdf]

Supplementary Materials

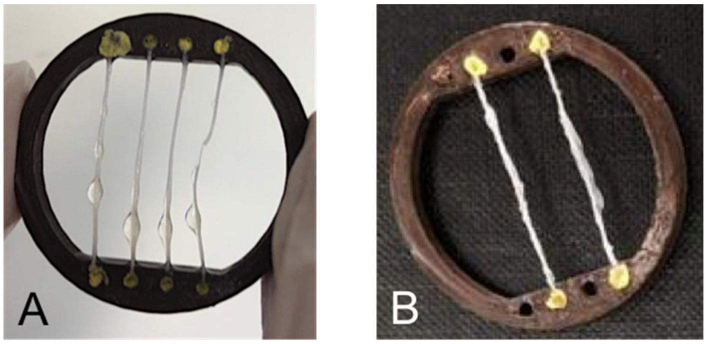

Figure S1. Collagen coating optimization. To obtain a uniform layer of collagen around the yarns, different formulations were tested. (A) Human collagen (Sigma-Aldrich, USA) + 10 U human transglutaminase (Zedira GmbH, Germany); (B) Bovine collagen (PureCol EZ Gel solution, 5074, Sigma-Aldrich, USA) + 10 U transglutaminase (Zedira GmbH, Germany). Yarns were coated with 100 μL of the collagen solutions and then incubated for 1 h at 37 °C under humidified conditions to allow gelation of the solution.

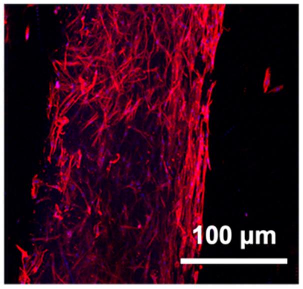

Figure S2. hASCs encapsulated in collagen-coated yarns after 3 days of culture. Cells were stained with Phalloidin-Rhodamine (cytoskeleton, red) and DAPI (nuclei, blue).

Table S1. Primer sequences used for RT-qPCR analysis.

Gene	Primer Sequence	Accession Number
**Housekeeping Genes**		
GAPDH	F: TGCACCACCAACTGCTTAGC<br>R: GGCATGGACTGTGGTCATGAG	NM_33197
GUSB	F: GATGTAGGTGGTGGGTGTCG<br>R: GCTCCGAATCACTATCGCCA	NM_000181.4
**Tenogenic-related Genes**		
MKX	F: TCGCACAGACACTCTGGAAAA<br>R: TGTTAAGGCCATAGCTGCGT	NM_173576.2
SCX	F: AGAACACCCAGCCCAAACAGAT<br>R: TCGCGGTCCTTGCTCAACTTT	NM_001080514.2
TNMD	F: CCGCGTCTGTGAACCTTTAC<br>R: CACCCACCAGTTACAAGGCA	NM_022144.2
**ECM-related Genes**		
COL1A1	F: GCCAAGACGAAGACATCCCA<br>R: GGCAGTTCTTGGTCTCGTCA	NM_000088.4
COL3A1	F: TTGGCATGGTTCTGGCTTCC<br>R: GCTGGCTACTTCTCGTG	NM_000090.3
DCN	F: CAGCATTCCTCAAGGTCTTCCT<br>R: GAGAGCCATTGTCAACAGCA	NM_001920.3
MMP1	F: ACCTGGAAAAATACTACAACCTGAA<br>R: TTCAATCCTGTACAGATGTGTT	NM_002421.3
MMP3	F: CACTCACAGACCTGACTCGG<br>R: AGTCAGGGGGAGGTCCATAG	NM_002422.4
TNC	F: ACTGCCAAGTTCACAACAGACC<br>R: CCCACAATGACTTCCTTGACTG	NM_002160.3
**Phenotypic Drift-related Genes**		
ACTA2	F: TAAGACGGGAATCCTGTGAAGC<br>R: TGTCCCATTCCCACCATCAC	NM_001141945.1
RUNX2	F: TTCCAGACCAGCAGCACTC<br>R: CAGCGTCAACACCATCATTC	NM_001024630
SOX9	F: TTCATGAAGATGACCGACGC<br>R: GTCCAGTCGTAGCCCTTGAG	NM_000346.3
